# Author Correction: c-Met activation leads to the establishment of a TGFβ-receptor regulatory network in bladder cancer progression

**DOI:** 10.1038/s41467-019-13095-4

**Published:** 2019-11-08

**Authors:** Wen Jing Sim, Prasanna Vasudevan Iyengar, Dilraj Lama, Sarah Kit Leng Lui, Hsien Chun Ng, Lior Haviv-Shapira, Eytan Domany, Dennis Kappei, Tuan Zea Tan, Azad Saei, Patrick William Jaynes, Chandra Shekhar Verma, Alan Prem Kumar, Mathieu Rouanne, Hong Koo Ha, Camelia Radulescu, Peter ten Dijke, Pieter Johan Adam Eichhorn, Jean Paul Thiery

**Affiliations:** 1 Institute of Molecular and Cell Biology, A*STAR, Singapore, 138672 Singapore; 2 Cancer Science Institute of Singapore, National University of Singapore, Singapore, 117599 Singapore; 3 Department of Cell and Chemical Biology and Oncode Institute, Leiden University Medical Center, 2333 ZC Leiden, The Netherlands; 4 Bioinformatics Institute (A*STAR), 30 Biopolis Street, 07-01 Matrix, Singapore, 138671 Singapore; 5 Department of Physics of Complex Systems, The Weizmann Institute of Science, Rehovot, Israel; 6 Department of Biochemistry, Yong Loo Lin School of Medicine, National University of Singapore, Singapore, 117596 Singapore; 7 Genome Institute of Singapore, A*STAR, Singapore, 138672 Singapore; 8 Department of Biological Sciences, National University of Singapore, 14 Science Drive, Singapore, 117543 Singapore; 9 School of Biological Sciences, Nanyang Technological University, 60 Nanyang Drive, Singapore, 637551 Singapore; 10 Department of Pharmacology, Yong Loo Lin School of Medicine, National University of Singapore, Singapore, 117597 Singapore; 11 Cancer Program, Medical Science Cluster, Yong Loo Lin School of Medicine, National University of Singapore, Singapore, Singapore; 12 Curtin Medical School, Faculty of Health Sciences, Curtin University, Perth, WA Australia; 13 Department of Urology, Hôpital Foch, Université Versailles-Saint-Quentin-en-Yvelines, Université Paris-Saclay, Suresnes, France; 14 INSERM Unit 1015, Laboratoire de Recherche Translationnelle en Immunologie (LRTI), Gustave Roussy, Université Paris-Saclay, Villejuif, France; 15 Department of Urology, Pusan National University Hospital, Pusan National University School of Medicine, Busan, 602−739 Korea; 16 Department of Pathology, Hôpital Foch, Université Versailles-Saint-Quentin-en-Yvelines, Université Paris-Saclay, Suresnes, France; 17 School of Pharmacy and Biomedical Sciences, Faculty of Health Sciences, Curtin University, Bentley, 6102 Australia; 18 Curtin Health Innovation Research Institute and Faculty of Health Sciences, Curtin University, Bentley, WA 6102 Australia; 19 Guangzhou Regenerative Medicine and Health, Guangdong Laboratory, 510530 Guangzhou, China

**Keywords:** Bladder cancer, Ubiquitylation

Correction to: *Nature Communications* 10.1038/s41467-019-12241-2, published online 25 September 2019.

The original version of this Article contained an error in the spelling of the author Azad Saei, which was incorrectly given as Azad Saie. This has now been corrected in both the PDF and HTML versions of the Article.

