# Supplementary material for: c-Met activation leads to the establishment of a TGFβ-receptor regulatory network in bladder cancer progression
Source: Nat Commun. 2019 Sep 25;10:4349. doi: 10.1038/s41467-019-12241-2 (PMC6761206; doi:10.1038/s41467-019-12241-2)
Supplement: Supplementary file 1 — Supplementary Information [file 41467_2019_12241_MOESM1_ESM.pdf]

Supplementary Information

c-Met activation leads to the establishment of a TGFβ-receptor regulatory network in bladder cancer progression

Sim et al.

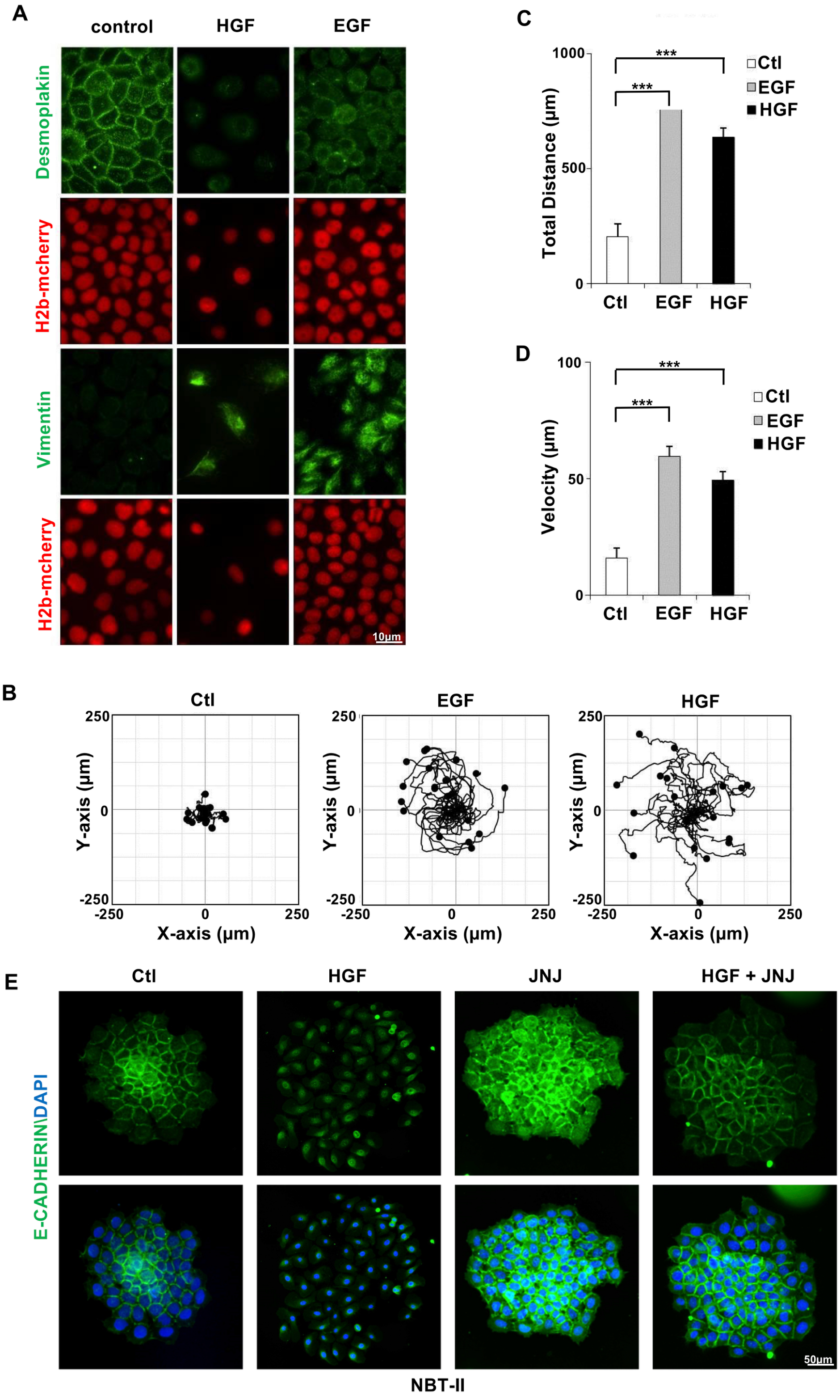

A
control
HGF
EGF
Desmoplakin
H2b-mcherry
Vimentin
H2b-mcherry
10µm
C
1000
500
0
Total Distance (µm)
***
***
Ctl
EGF
HGF
Ctl
EGF
HGF
D
100
50
0
Velocity (µm)
***
***
Ctl
EGF
HGF
Ctl
EGF
HGF
B
Ctl
EGF
HGF
250
0
-250
Y-axis (µm)
-250
0
250
X-axis (µm)
E
Ctl
HGF
JNJ
HGF + JNJ
E-CADHERIN\DAPI
50µm
NBT-II

Supplementary Figure 1

(A) NBT-II carcinoma cells were treated with HGF or EGF and immunofluorescent stained for Desmoplakin (1st panel) and Vimentin (3rd panel). Scale bars: 10 μm. (**B**) The effect of HGF or EGF on individual cell tracks recorded at each time point for 24 hours. The movement of NBT-II cells become increasingly erratic over 24 hours following the addition of EGF or HGF. **(C)** HGF and EGF increased the total distance of NBT-II cells over 24 hours. Bars represent mean ± SD of three independent experiments. A 2-tailed Student's *t* test compares the treated populations, ***P<0.001. **(D)** HGF increased the velocity of NBT-II cells over 24 hours. Bars represent mean ± SD of three independent experiments. A 2-tailed Student's *t* test compares the treated populations, ***P<0.001. **(E)** NBT-II carcinoma cells were treated with HGF in the presence or absence of JNJ38877605 and immunofluorescent stained for E-cadherin and DAPI. Scale bars: 50 μm.

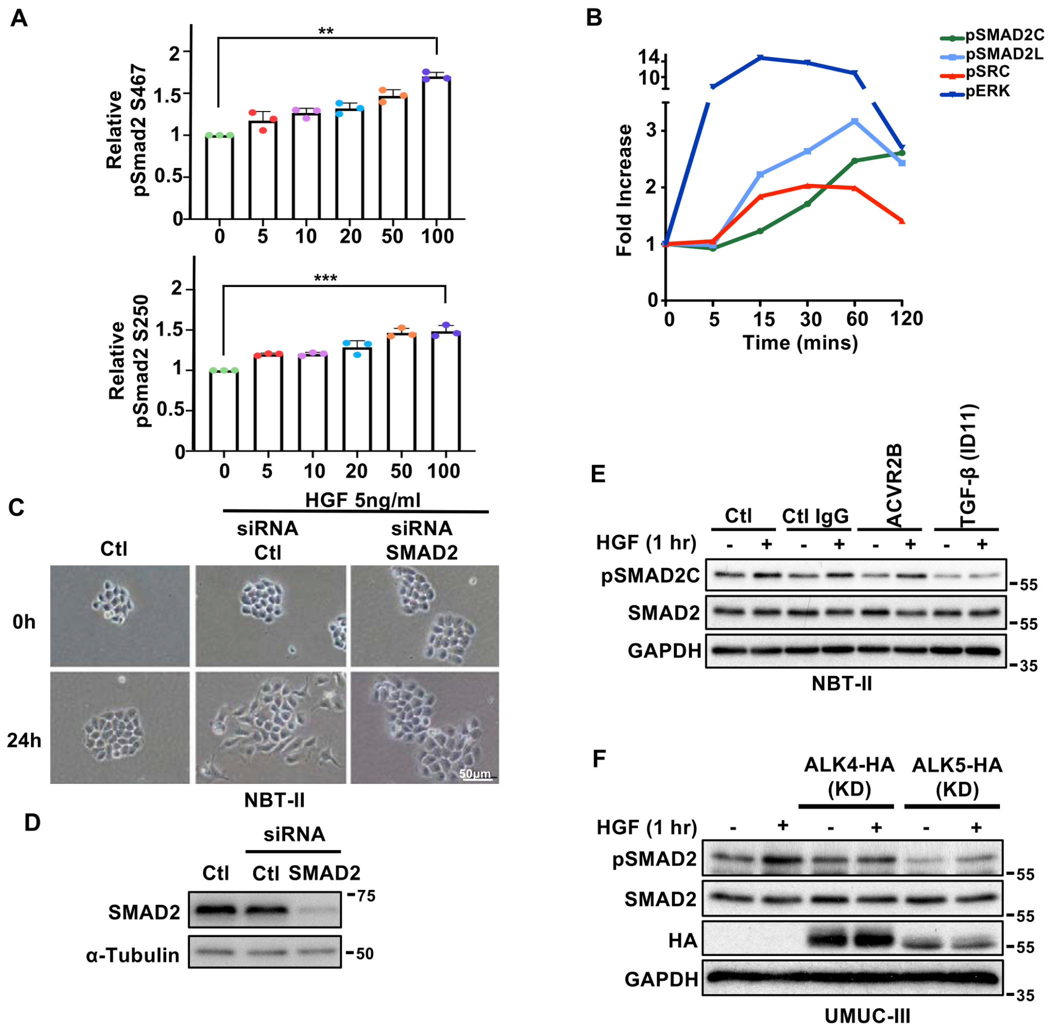

Supplementary Figure 2

(A) Quantification of Figure 2B comparing phospho-proteins to corresponding total proteins. Density was evaluated using IMAGE J. Bars represent mean ± SD of three independent experiments. A 2-tailed Student's *t* test compares the treated populations, *P<0.05. (**B**) Quantification of Figure 2C comparing phospho-proteins to corresponding total proteins. Density was evaluated using IMAGE J. Data represents the mean from two independent experiments. **(C-D)** Phase contrast images **(C)** and western blot analysis **(D)** of NBT-II cells transfected with either control siRNA or SMAD2 siRNA under HGF induction for 24 hours. Whole cell extracts were probed with the indicated antibodies. Scale bars: 50 µm. (**E**) Western blot analysis of NBT-II cells treated with 5ng/ml of HGF in the absence or presence activin ligand trap ACVR2B-Fc or pan-TGFβ1,2,3 neutralizing antibodies (pre-treatment for 1 hour). Lysates were collected and probed with indicated antibodies. (**F**) NBT-II cells were transfected as indicated with kinase dead mutants of ALK4 or ALK5 and treated with 5 ng/ml of HGF. Lysates were collected and probed with indicated antibodies.

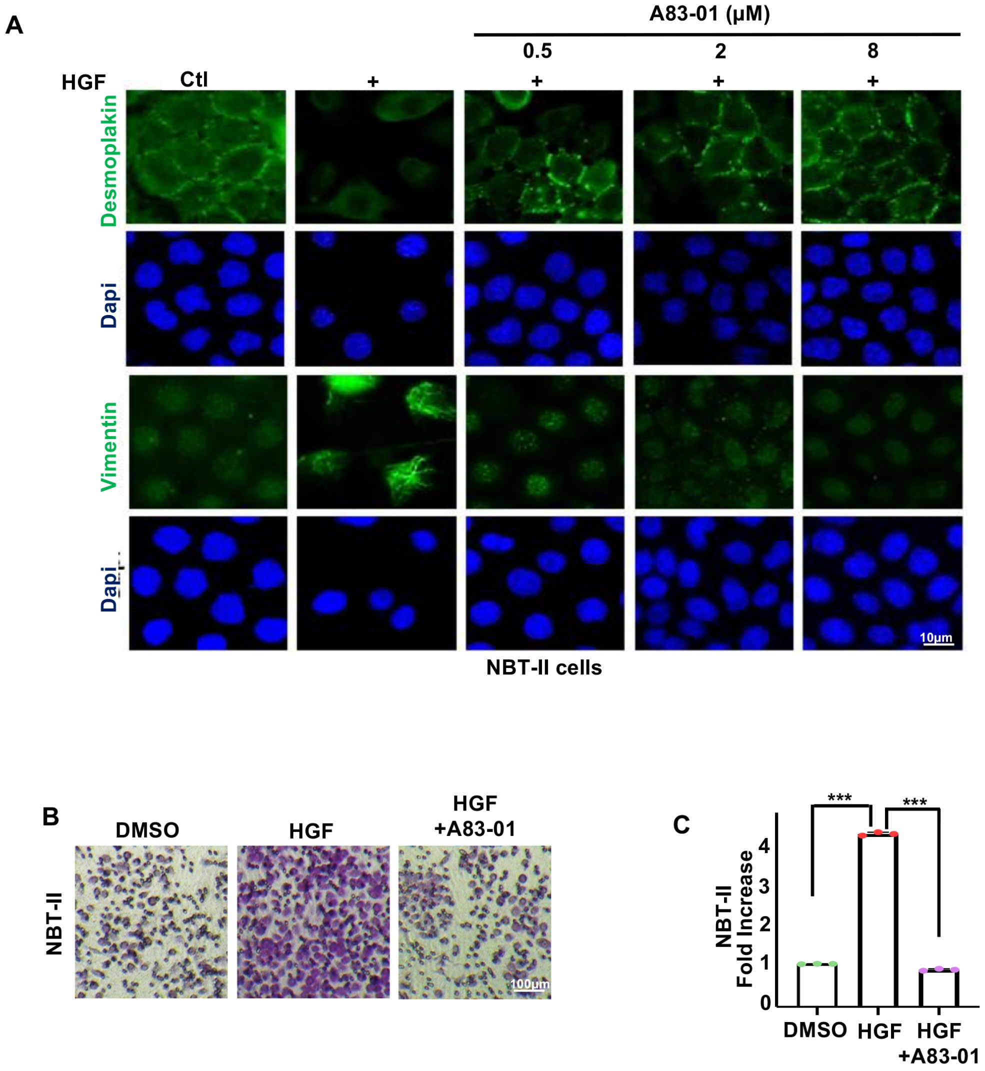

Supplementary Figure 3

(A) NBT-II carcinoma cells were treated with increasing doses (0.5 µM, 2 µM, 8 µM) of A83-01 and immunofluorescent stained for Desmoplakin (1st panel) and Vimentin (3rd panel). Scale bars: 10 µm. **(B)** Transwell assay of NBT-II cells treated with HGF -/+ A83-01 (8 µM) for 16 hours prior to fixation and crystal violet staining. Scale bars: 100 µm. **(C)** The graph represents the fold change of the number of migrated cells taken from four different random fields from panel. Data are mean ± SD of triplicate samples from a representative experiment performed three times. Student's *t* test compares the treated populations, ***P<0.001.

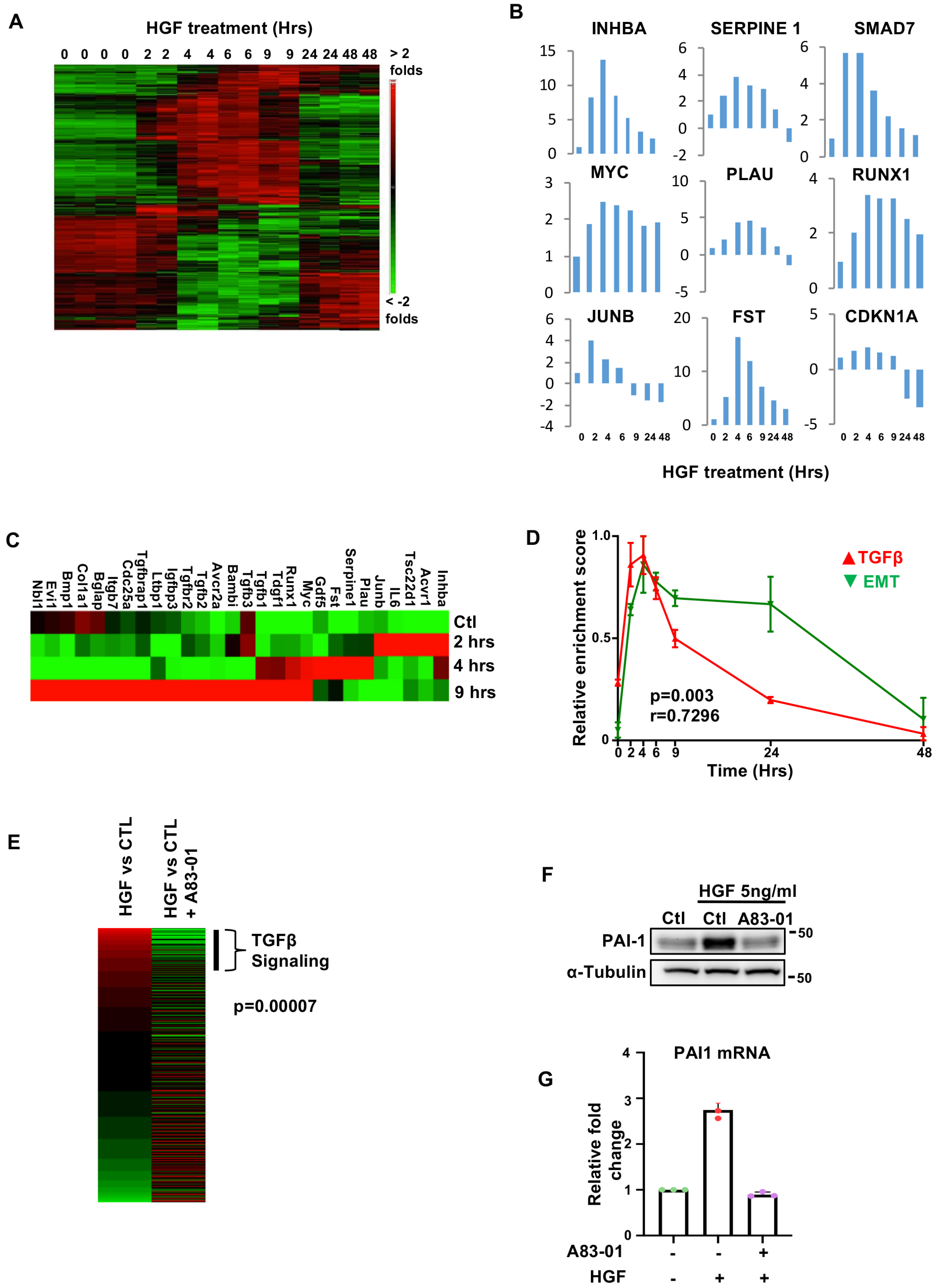
A
HGF treatment (Hrs)
0 0 0 0 2 2 4 4 6 6 9 9 24 24 48 48
> 2 folds
< -2 folds
B
INHBA
SERPINE 1
SMAD7
MYC
PLAU
RUNX1
JUNB
FST
CDKN1A
0 2 4 6 9 24 48
HGF treatment (Hrs)
C
Nbl1
Evi1
Bmp1
Col1a1
Bglap
Itgb7
Cdc25a
Tgfbrap1
Ltbp1
Igfbp3
Tgfbr2
Tgfb2
Avcr2a
Bambi
Tgfb3
Tgfb1
Tdgf1
Runx1
Myc
Gdf5
Fst
Serpine1
Plau
Junb
IL6
Tsc22d1
Acvr1
Inhba
Ctl
2 hrs
4 hrs
9 hrs
D
Relative enrichment score
▲TGFβ
▼EMT
p=0.003
r=0.7296
Time (Hrs)
E
HGF vs CTL
HGF vs CTL + A83-01
TGFβ Signaling
p=0.00007
F
HGF 5ng/ml
Ctl
Ctl
A83-01
PAI-1
α-Tubulin
-50
-50
G
PAI1 mRNA
Relative fold change
A83-01
HGF

Supplementary Figure 4

(A) The unsupervised clustering of the mean intensities of samples treated without growth factor of with growth factor for 2, 4, 6, 9, 24 and 48 hours. **(B)** Bar chart displaying relative fold change of selected TGFβ target genes from **(A)** at different time-points compared to 0 hr (control). **(C)** Heat Map of 28 altered TGFβ target genes extrapolated from RT^2 TGFβ profiler array performed in NBT-II cells treated with HGF at indicated time points. (**D**) Graph displays relative enrichment score of HGF induced TGFβ signalling compared to either MSigb v5.0 Hallmark TGFβ or EMT signatures. The p-value is computed by Spearman correlation coefficient test.**(E)** The unsupervised clustering of the mean intensities in the two repeats of each condition:1) DMSO (drug vehicle) without (GF) growth factor versus DMSO+HGF and 2) DMSO+HGF versus A83-01+HGF. The addition of A83-01 reversed the effect of HGF for a group of 280 genes; A83-01 failed to revert a group of 177 HGF driven genes back to control conditions; and 99 genes were the side-effects of the drug. The p-value is computed by hypergeometric test. **(F)** NBT-II cells treated with HGF 5ng/ml for 2 hours in the absence or presence of A83-01(8 μM). Whole cell extracts were probed with the indicated antibodies. (**G**) Bar chart display relative fold change of PAI-1 mRNA expression in cells treated with or without HGF and A83-01.

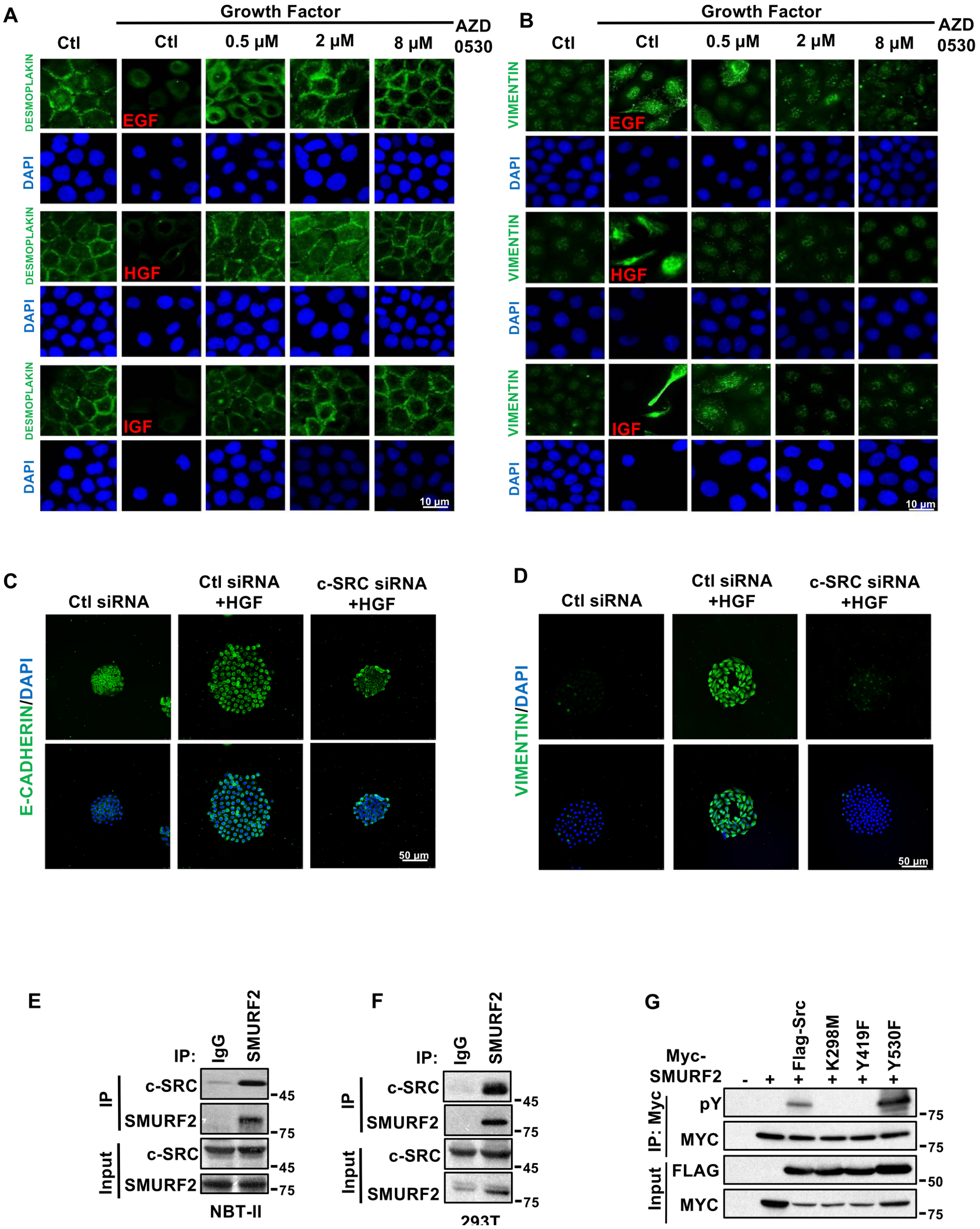
A
Growth Factor
AZD 0530
Ctl
Ctl
0.5 μM
2 μM
8 μM
DESMOPLAKIN
DAPI
EGF
HGF
IGF
10 μm
B
Growth Factor
AZD 0530
VIMENTIN
DAPI
EGF
HGF
IGF
10 μm
C
Ctl siRNA
Ctl siRNA +HGF
c-SRC siRNA +HGF
E-CADHERIN/DAPI
50 μm
D
Ctl siRNA
Ctl siRNA +HGF
c-SRC siRNA +HGF
VIMENTIN/DAPI
50 μm
E
IP:
IgG
SMURF2
IP
Input
c-SRC
SMURF2
45
75
NBT-II
F
IP:
IgG
SMURF2
c-SRC
SMURF2
293T
G
Myc-SMURF2
-
+
+ Flag-Src
+ K298M
+ Y419F
+ Y530F
IP: Myc
Input
pY
MYC
FLAG
MYC
75
50

Supplementary Figure 5

(A-B) NBT-II carcinoma cells were treated with either EGF, HGF, or IGF along with increasing doses (0.5 µM, 2 µM, 8 µM) of AZD0530 and immunofluorescent stained for Desmoplakin **(A)** and Vimentin **(B).** Scale bars: 10 µm. (**C**) NBT-II carcinoma cells were treated with HGF in the presence or absence of siRNA targeting c-SRC and immunofluorescent stained for E-cadherin. Scale bars: 50 µm. (**D**) NBT-II carcinoma cells were treated with HGF in the presence or absence of siRNA targeting c-SRC and immunofluorescent stained for Vimentin. Scale bars: 50 µm. (**E**) NBT-II cells lysed and immunoprecipitated with anti-SMURF2 affinity resin. Whole cell extracts were probed with indicated antibodies. **(F)** 293T cells lysed and immunoprecipitated with anti-SMURF2 affinity resin. Whole cell extracts were probed with indicated antibodies. (**G**) 293T cells were transfected as indicated with MYC tagged SMURF2 and c-SRC WT, dominant negative mutants K298M, Y419F or dominant active mutant Y530F. After 48hrs cells were lysed and immunoprecipitated with anti-MYC affinity resin. Whole cell extracts were probed with the indicated antibodies.

A

SMURF2 phosphorylation site	Position	SILAC ratio forward	SILAC ratio reverse
VVVDG**S**GQCHSTDTVK	44	0.86	1.17
THLH**T**PPDLPEGYEQR	249	0.60	0.66
V**Y**FVDHNNR	314	14.11	n.d.
QEL**S**QQQPQAGHCR	384	n.d.	0.68
DDIY**T**LQINPDSAVNPEHLSYFHFVGR	466	n.d.	n.d.
SMURF2 unmodified		0.98	0.98

B

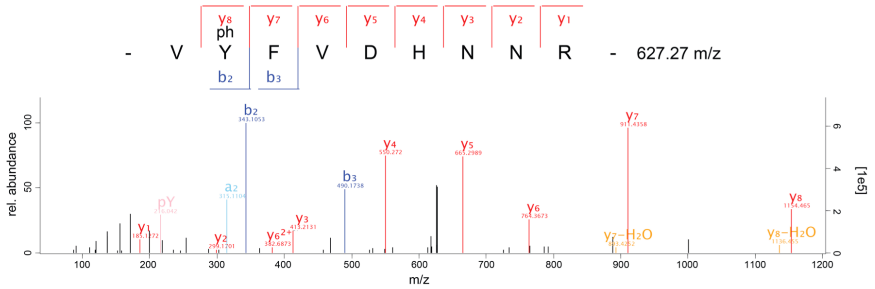

Supplementary Figure 6

(A) Table representing identified phosphorylation sites by Mass Spectrometry and SILAC ratios in the presence and absence of co-transfected c-SRC. **(B)** HPLC/MS/MS spectrum of phosphopeptide VY(P)FVDHNNR. The notations b_n^{Δ} denotes the corresponding b_n ions minus H_3PO_4, which serve to confirm the phosphopeptide. Mass accuracy: ±1 Da (monoisotopic mass).

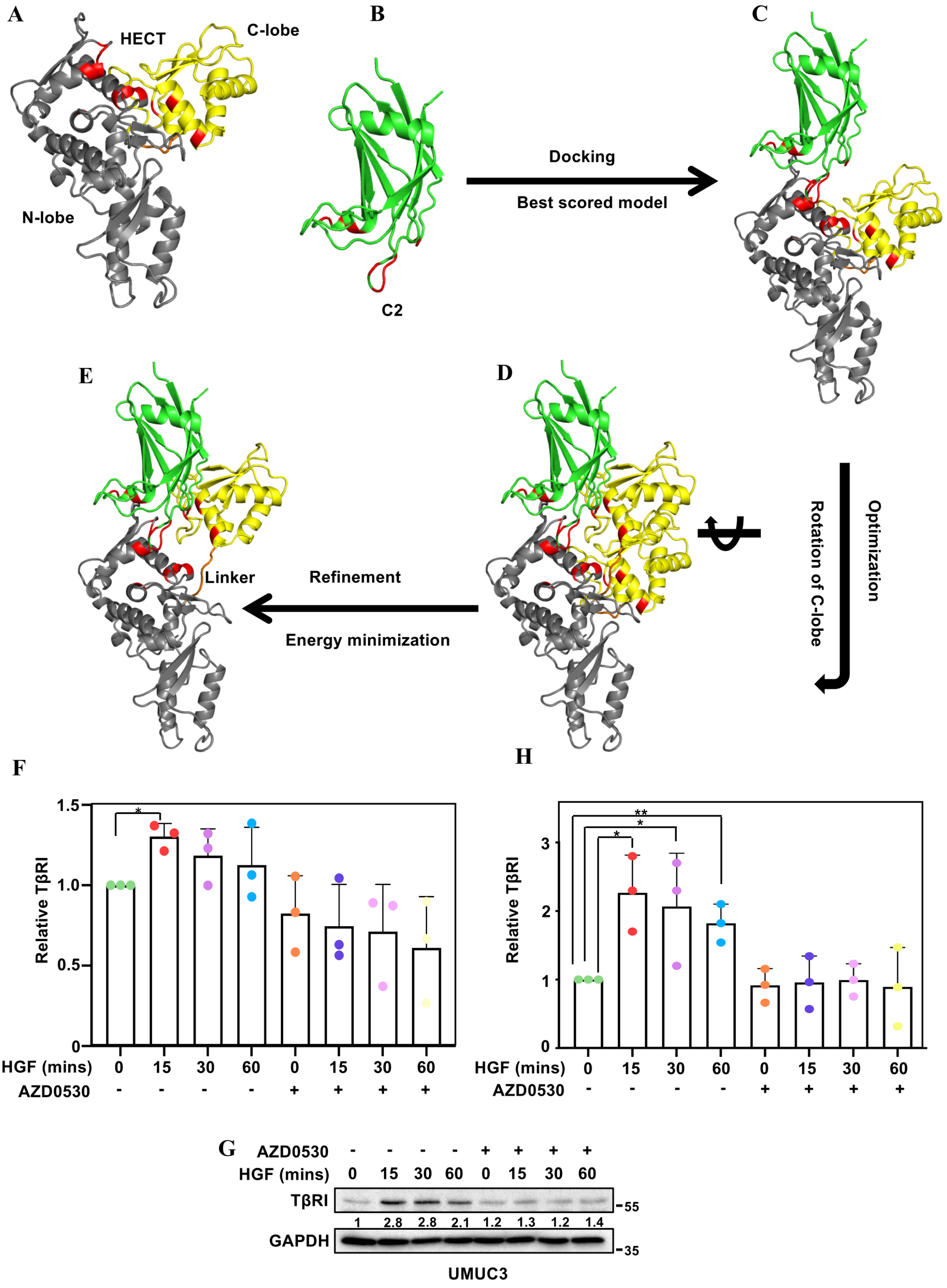
A
HECT
C-lobe
N-lobe
B
C2
Docking
Best scored model
C
E
D
Linker
Refinement
Energy minimization
Rotation of C-lobe
Optimization
F
Relative TβRI
HGF (mins) 0 15 30 60 0 15 30 60
AZD0530 - - - - + + + +
H
Relative TβRI
HGF (mins) 0 15 30 60 0 15 30 60
AZD0530 - - - - + + + +
G
AZD0530 - - - - + + + +
HGF (mins) 0 15 30 60 0 15 30 60
TβRI
1 2.8 2.8 2.1 1.2 1.3 1.2 1.4
GAPDH
-55
-35
UMUC3

Supplementary Figure 7

(A, B) Ribbon representation of HECT and C2 domain structures of SMURF2 (PDB IDs: 1ZJD and 2JQZ) respectively. The N- lobe, C-lobe and the connecting linker of HECT domains are shown in grey, yellow and orange colour respectively. The active and passive residues defined for docking are coloured in red for both the domains. **(C)** Best scored model from the clusters of structures generated from the docking of the two domains. **(D)** Optimized model of the complex obtained by rigid body rotation of the C-lobe in HECT domain around the linker. **(E)** Final refined docked complex structure of HECT-C2 domains of SMURF2 obtained after energy minimization. (**F**) Quantification of Figure 6B comparing TβRI to corresponding total proteins. Density was evaluated using IMAGE J. Bars represent mean ± SD of three independent experiments. A 2-tailed Student's *t* test compares the treated populations, *P<0.05. (**G**) UMUC3 cells treated with HGF (5 ng/ml) and/or AZD0530 (1 μM) lysed at the indicated time points. Whole cell extracts were probed with the indicated antibodies**. (H)** Quantification of Figure S7G comparing TβRI to corresponding total proteins. Density was evaluated using IMAGE J. Bars represent mean ± SD of three independent experiments. A 2-tailed Student's *t* test compares the treated populations, *P<0.05, **P<0.01, ***P<0.001.

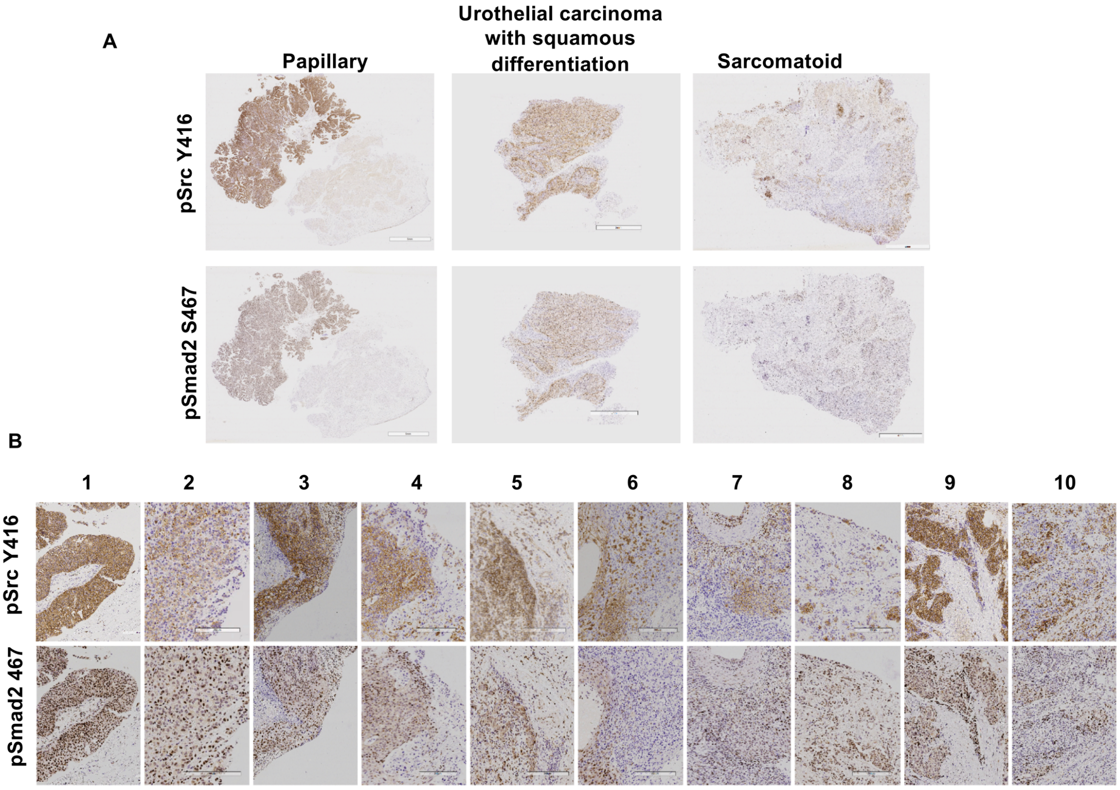

C

	Gender	Age	Type	Stage	Nodes
1	M	72	Papillary	pT1	N0
2	M	73	Papillary	pT3b	N0
3	M	77	Urothelial carcinoma with squamous differentiation	pT3b	N0
4	M	80	Urothelial carcinoma with squamous differentiation (Partial)	pT3b	N0
5	F	70	Urothelial carcinoma with squamous differentiation (Partial)	pT4b	N2
6	M	45	Urothelial carcinoma with squamous differentiation	pT3a	N0
7	F	83	Urothelial carcinoma with squamous differentiation	pT4a	N0
8	F	74		pT4a	N2
9	M	85	Neuro endocrine	pT3b	N2
10	F	62	Sarcomatoid (60%)	pT3b	N0

Supplementary Figure 8

(A) IHC of papillary, urothelial carcinoma with squamous differentiation, neuroendocrine, and sarcomatoid bladder tumour samples for p-c-SRC and pSMAD2C. Representative images are displayed. Original magnification 400X. Scale bars: 5 mm. **(B)** IHC analysis of p-c-SRC Y416 and pSMAD2C in individual bladder tumour sections. Top panel: IHC stain for p-c-SRC shows distinct membrane expression; bottom panel: IHC stain for pSMAD2C shows nuclear expression. Correlation of p-c-SRC and pSMAD2 expression in papillary (1,2), urothelial carcinoma with squamous differentiation (3-8), neuro endocrine (9), and sarcomatoid (10). Scale bars: 500 µm. (**C**) Table representing individual characteristics of patients represented in figure **B**.

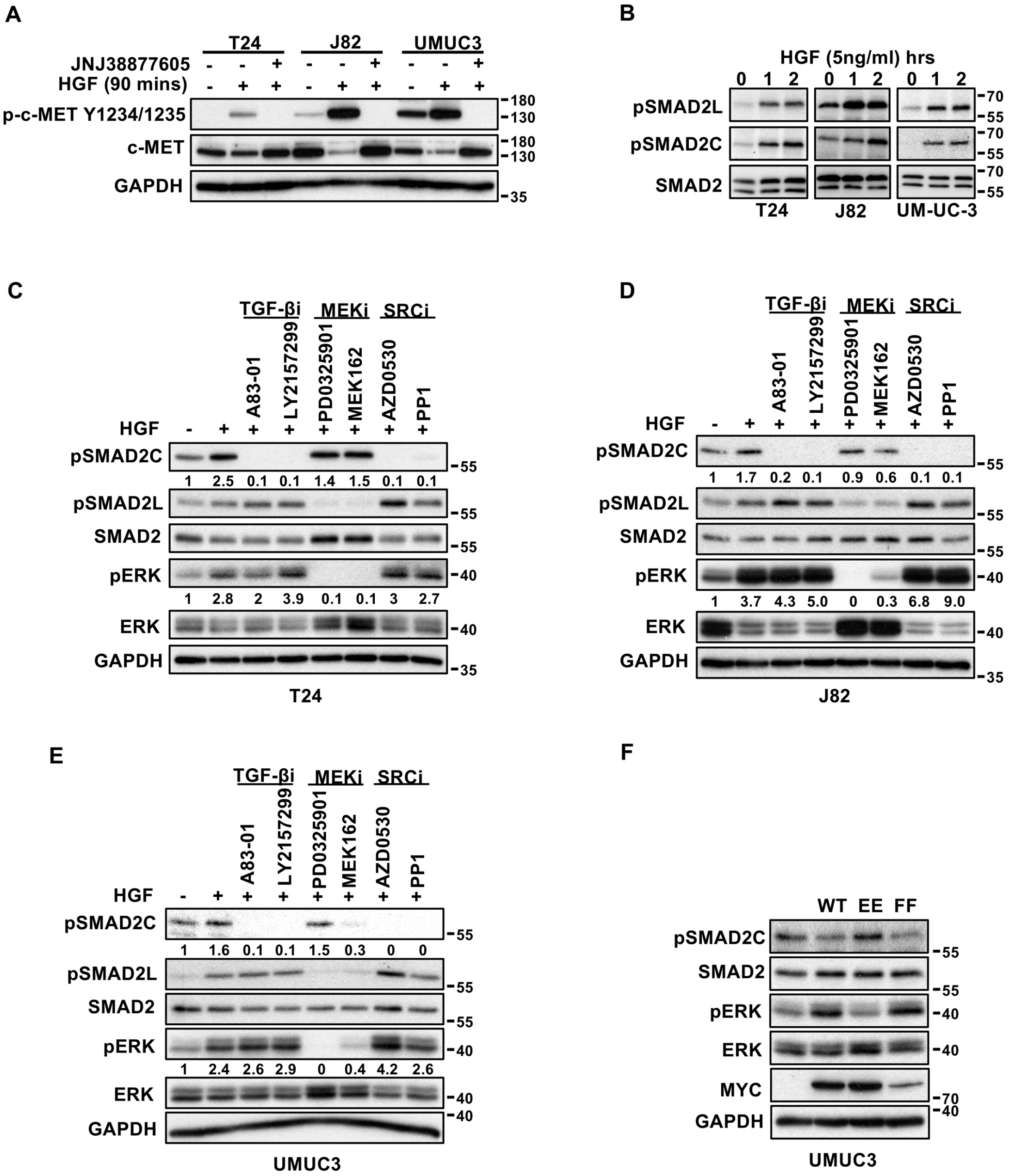

Supplementary Figure 9

(A) Western blot analysis of T24, J82, and UMUC3 cells treated with 5 ng/ml of HGF in the absence or presence of c-Met inhibitor JNJ38877605. Lysates are probed with indicated antibodies. (**B)** Western blot analysis of T24, J82, and UMUC3 cells treated with 5 ng/ml of HGF. Lysates are probed with indicated antibodies. (**C-E**) Western blot analysis of T24 (**C**), J82 (**D**), or UMUC3 (**E)** cells treated with 5 ng/ml of HGF and A83-01 (8 μM), or LY2157299 (1 μM), PD0325901 (1 μM), MEK162 (1 μM), AZD0530 (1 μM), and PP1 (1 μM). Lysates were collected at 90 minutes and probed with indicated antibodies. (**F**) UMUC3 transfected as indicated. Lysates were probed with the indicated antibodies.

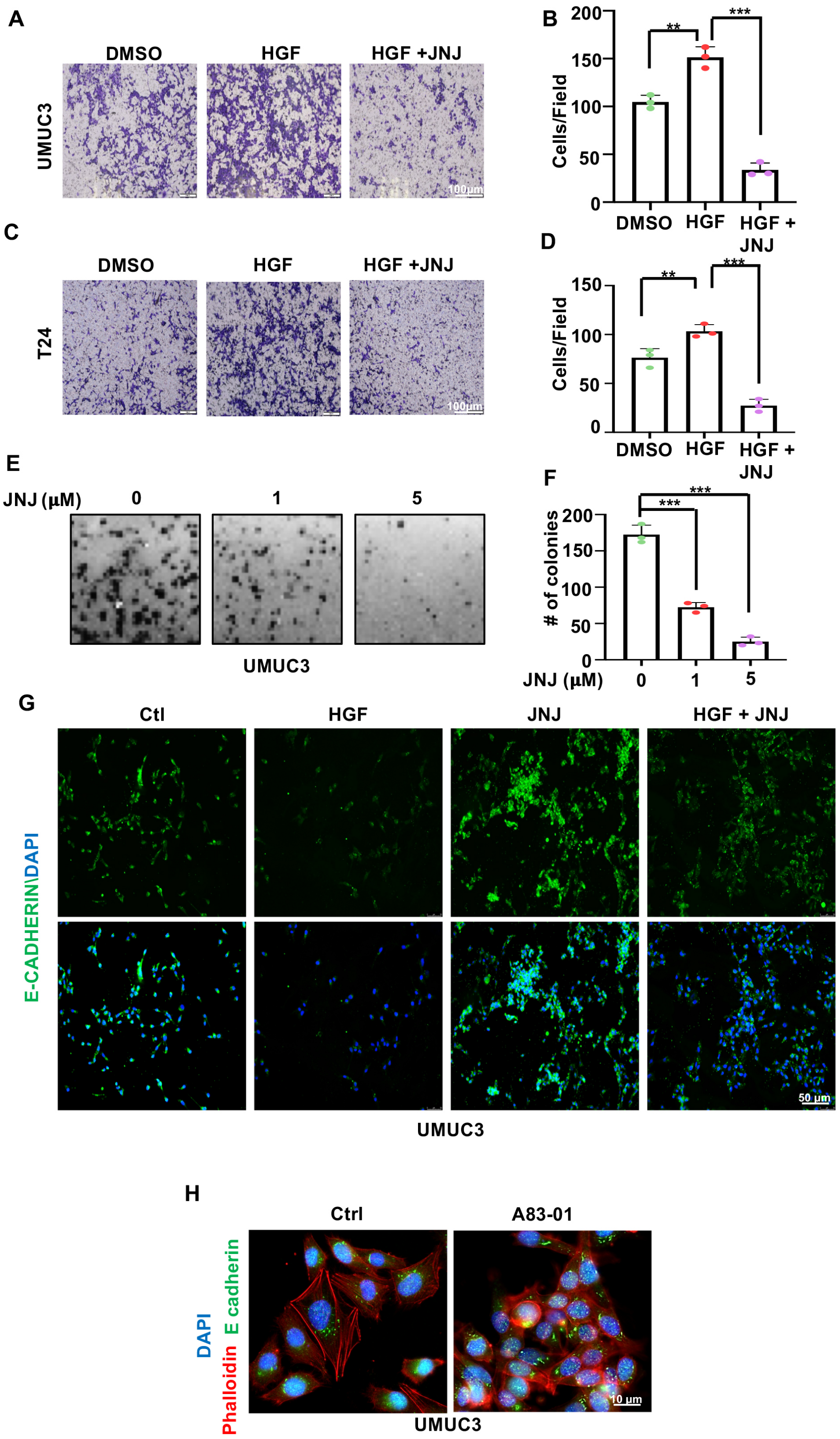
A
DMSO
HGF
HGF +JNJ
UMUC3
100μm
B
200
150
100
50
0
Cells/Field
**
***
DMSO
HGF
HGF + JNJ
C
DMSO
HGF
HGF +JNJ
T24
100μm
D
150
100
50
0
Cells/Field
**
***
DMSO
HGF
HGF + JNJ
E
JNJ (μM)
0
1
5
UMUC3
F
200
150
100
50
0
# of colonies
***
***
JNJ (μM)
0
1
5
G
Ctl
HGF
JNJ
HGF + JNJ
E-CADHERIN\DAPI
50 μm
UMUC3
H
Ctrl
A83-01
Phalloidin E cadherin DAPI
10 μm
UMUC3

Supplementary Figure 10

A) Transwell assay of UMUC3 cells treated with HGF (5 ng/ml) and JNJ38877605 (4 μM) or the combination for 16 hours prior to fixation and crystal violet staining. Scale bars: 100 μm. **(B)** Graph represents average number of migrated cells taken from four different random fields from panel. Data are mean ± SD of triplicate samples from a representative experiment performed three times. Student's *t* test compares the treated populations, **P<0.01, ***P<0.001. **(C)** Transwell assay of T24 cells treated with HGF (5 ng/ml) and JNJ38877605 (4 μM) or the combination for 16 h prior to fixation and crystal violet staining. Scale bars: 100 μm. **(D)** Graph represents average number of migrated cells taken from four different random fields from panel. Data are mean ± SD of triplicate samples from a representative experiment performed three times. Student's *t* test compares the treated populations, **P<0.01, ***P<0.001. **(E)** Soft agar growth assay of UMUC3 cells treated with either JNJ38877605 (4 μM). (**F)** Graph represents average number of colonies taken from four different random fields from panel. Data are mean ± SD of triplicate samples from a representative experiment performed three times. Student's *t* test compares the treated populations, ***P<0.001. (**G**) UMUC3 bladder carcinoma cells were treated with HGF (5 ng/ml) and/or JNJ38877605 (4 μM) and immunofluorescent stained for E-cadherin (green). Scale bars: 50 μm. (**H**) UMUC3 bladder carcinoma cells were treated with A83-01 (5 μM) and immunofluorescent stained for Phalloidin (red), E-cadherin (green). Scale bars: 10 μm.

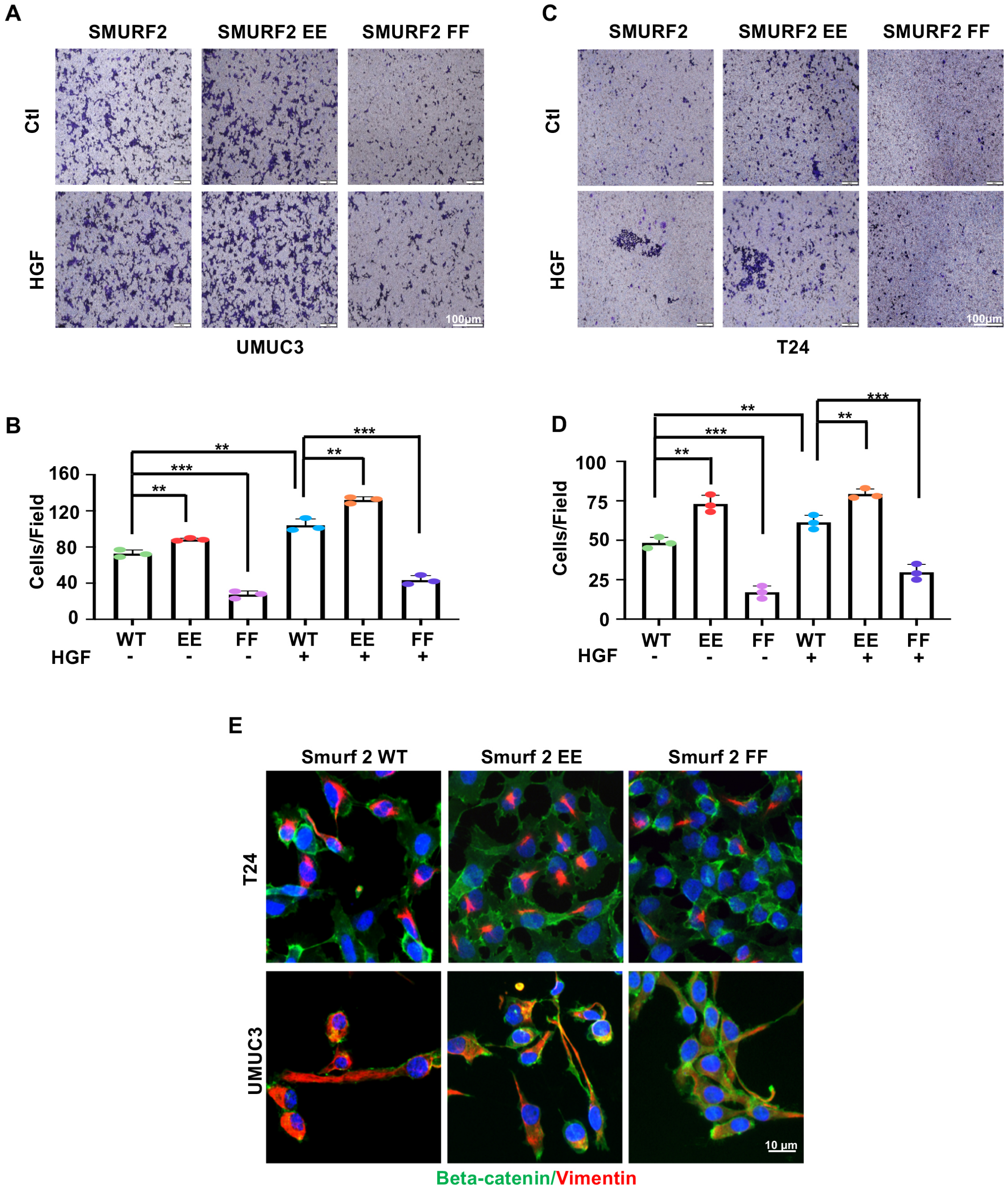
A
SMURF2
SMURF2 EE
SMURF2 FF
Ctl
HGF
100μm
UMUC3
C
SMURF2
SMURF2 EE
SMURF2 FF
Ctl
HGF
100μm
T24
B
Cells/Field
160
120
80
40
0
WT
EE
FF
WT
EE
FF
HGF
-
-
-
+
+
+
**
***
**
**
***
D
Cells/Field
100
75
50
25
0
WT
EE
FF
WT
EE
FF
HGF
-
-
-
+
+
+
**
***
**
***
**
E
Smurf 2 WT
Smurf 2 EE
Smurf 2 FF
T24
UMUC3
10 μm
Beta-catenin/Vimentin

Supplementary Figure 11

Transwell assay of UMUC3 cells transfected with either SMURF2, SMURF2(EE), or SMURF2(FF) in the presence or absence of HGF (5 ng/ml) for 16 hours prior to fixation and crystal violet staining. Scale bars: 100 µm. **(B)** Graph represents average number of migrated cells taken from four different random fields from panel **A**. Data are mean ± SD of triplicate samples from a representative experiment performed three times. Student's *t* test compares the treated populations, * P<0.05, ** P<0.01 ***P<0.001. **(C)** Transwell assay of T24 cells transfected with either SMURF2, SMURF2(EE), or SMURF2(FF) in the presence or absence of HGF (5 ng/ml) for 16 hours prior to fixation and crystal violet staining. Scale bars: 100 µm. **(D)** Graph represents average number of migrated cells taken from four different random fields from panel **C**. Data are mean ± SD of triplicate samples from a representative experiment performed three times. Student's *t* test compares the treated populations, * P<0.05, ** P<0.01 ***P<0.001. **(E)** T24 (top panel) or UMUC3 (bottom panel) bladder carcinoma cells were treated with transfected as shown and treated with HGF (5 ng/ml) and immunofluorescent stained for β-catenin (green) and Vimentin (red). Scale bars: 10 µm.

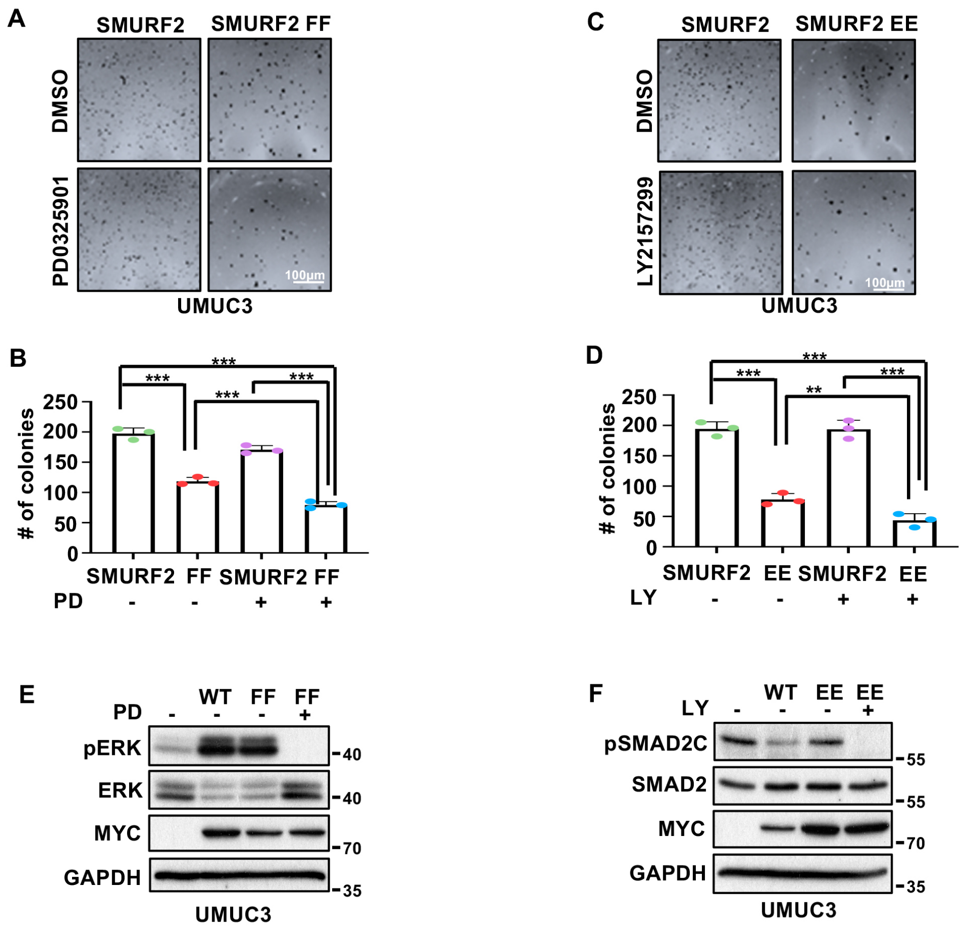

Supplementary Figure 12

(**A**) Soft agar growth assay of UMUC3 cells transfected with either SMURF2, or SMURF2(FF) in the presence or absence of PD0325901(1 µM). Scale bars: 100 µm. **(B)** Graph represents average number of colonies taken from four different random fields from panel **A**. Data are mean ± SD of triplicate samples from a representative experiment performed three times. Student's *t* test compares the treated populations, * P<0.05, ** P<0.01 ***P<0.001. **(C)** Soft agar growth assay of UMUC3 cells transfected with either SMURF2, or SMURF2(EE) in the presence or absence of LY2157299(1 µM). Scale bars: 100 µm. **(D)** Graph represents average number of colonies taken from four different random fields from panel **C**. Data are mean ± SD of triplicate samples from a representative experiment performed three times. Student's *t* test compares the treated populations, * P<0.05, **P<0.01, *** P<0.001. **(E)** Western blot analysis of UMUC3 cells transfected as indicated in the absence or presence of PD0325901(1 µM). Lysates are probed with indicated antibodies. **(F)** Western blot analysis of UMUC3 cells transfected as indicated in the absence or presence of LY2157299(1 µM). Lysates are probed with indicated antibodies.

A

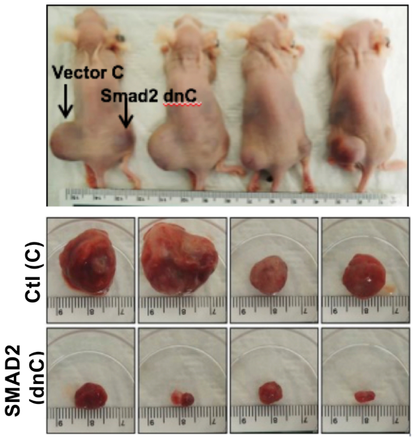

B

	Ctl (C)	SMAD2 (dnC)
Incidence	4/4 (100%)	4/4 (100%)

C

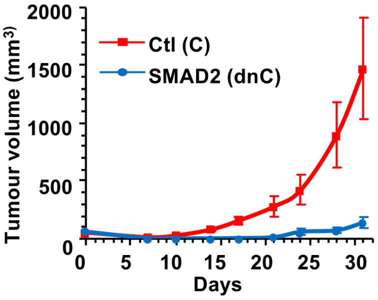

Supplementary Figure 13

(A) UMUC3 cells stably transfected with control vector or SMAD2 C-terminal mutant were subcutaneously implanted into the flank of mice and monitored for one month. (**B**) Table displays tumour incidence of UMUC3 WT and UMUC3 SMAD2 C-terminal mutant. **(C)** Graph displays tumour volume of WT and UM-UC-3 SMAD2 C-terminal mutant implanted over one month.

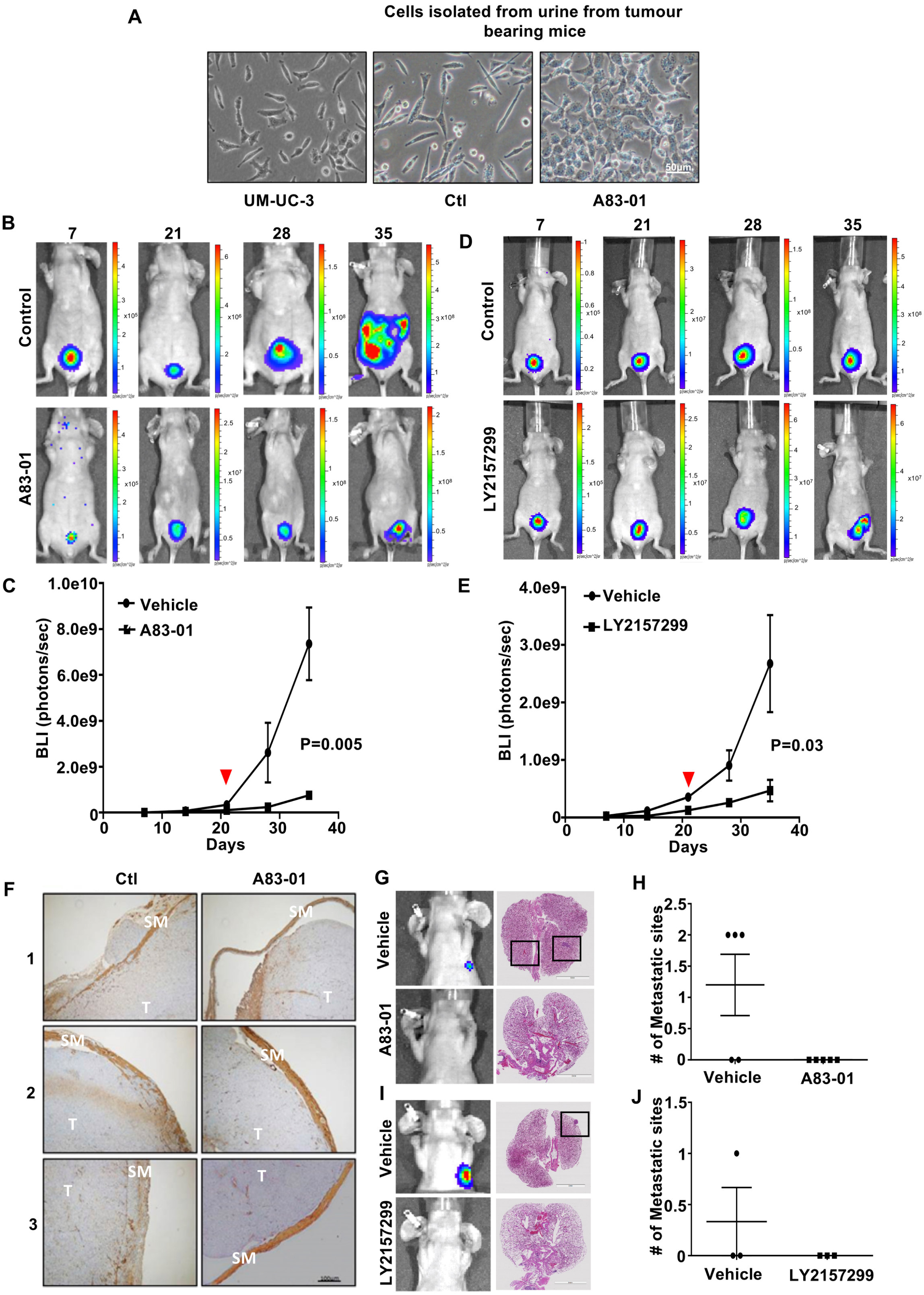
A
Cells isolated from urine from tumour bearing mice
50µm
UM-UC-3
Ctl
A83-01
B
7
21
28
35
Control
A83-01
D
Control
LY2157299
C
BLI (photons/sec)
Vehicle
A83-01
P=0.005
Days
E
Vehicle
LY2157299
P=0.03
F
Ctl
SM
T
1
2
3
G
Vehicle
H
# of Metastatic sites
I
J

Supplementary Figure 14

(A) Phase contrast images of UM-UC3 cells (Left), urine isolated cells from mice treated with drug vehicle (Middle) or A83-01 (Right). (**B**) Bioluminescent images of mice injected luciferase tagged UMUC3 cells intraluminally injected in the bladder. Daily administration of vehicle control only (Control), A83-01 (50 mg/kg), started when bioluminescence intensity reaches ~ $5e^8$ photons/second. Tumour volumes were measured twice a week (n=5). Scale bars = p/sec/cm^2/sr. (**C**) Graph of data extrapolated from **B**, BLI = photons/sec, p=0.005. (**D**) Bioluminescent images of mice injected luciferase tagged UMUC3 cells intraluminally injected in the bladder. Daily administration of vehicle control only (Control), LY2157299 (80 mg/kg), started when bioluminescence intensity reaches ~ $5e^8$ photons/second. Tumour volumes were measured twice a week (n=3). Scale bars = p/sec/cm^2/sr. (**E**) Graph of data extrapolated from **D**, BLI = photons/sec, p=0.03. (**F**) Immunohistochemistry of α-SMA expression, a marker for bladder wall, in bladder tumour of mice treated with drug vehicle or A83-01. Scale bars: 100 μm. (**G**) Bioluminescent images of mice of lung metastasis from **B** (left panel) and representative tissue sections highlighting tumour formation (right panel). (**H**) Graph of data extrapolated from **G**, (n=5). (**I**) Bioluminescent images of mice of lung metastasis from **D** (left panel) and representative tissue sections highlighting tumour formation (right panel). (**J**) Graph of data extrapolated from **I**, (n=3).

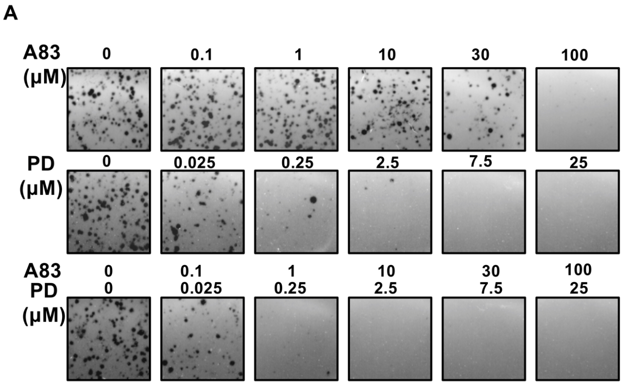

Supplementary Figure 15
(A) Soft agar growth assay of UMUC3 cells treated with either A83-01, PD0325901, or both.
