# Supplementary material for: c-Met activation leads to the establishment of a TGFβ-receptor regulatory network in bladder cancer progression
Source: Nat Commun. 2019 Sep 25;10:4349. doi: 10.1038/s41467-019-12241-2 (PMC6761206; doi:10.1038/s41467-019-12241-2)
Supplement: Supplementary file 3 — Description of Additional Supplementary Files [file 41467_2019_12241_MOESM3_ESM.pdf]

Description of Additional Supplementary Files

File Name: Supplementary Data 1
Description: Quantification of protein phosphorylation following HGF treatment.

File Name: Supplementary Data 2
Description: Quantification of TβR/BMPR signalling pathway profiler by qRT-PCR.

File Name: Supplementary Data 3
Description: Enrichr pathway enrichment analysis.

File Name: Supplementary Data 4
Description: Quantification of EMT score, SMURF2, MET, and HGF expression in bladder cancer cell lines.
