# Supplementary material for: c-Met activation leads to the establishment of a TGFβ-receptor regulatory network in bladder cancer progression
Source: Nat Commun. 2019 Sep 25;10:4349. doi: 10.1038/s41467-019-12241-2 (PMC6761206; doi:10.1038/s41467-019-12241-2)
Supplement: Supplementary file 8 — Reporting Summary [file 41467_2019_12241_MOESM8_ESM.pdf]

nature research

Corresponding author(s): Pieter Eichhorn, Jean Paul Thiery

Last updated by author(s): Aug 6, 2019

Reporting Summary

Nature Research wishes to improve the reproducibility of the work that we publish. This form provides structure for consistency and transparency in reporting. For further information on Nature Research policies, see Authors & Referees and the Editorial Policy Checklist.

Please do not complete any field with "not applicable" or n/a. Refer to the help text for what text to use if an item is not relevant to your study.
For final submission: please carefully check your responses for accuracy; you will not be able to make changes later.

Statistics

For all statistical analyses, confirm that the following items are present in the figure legend, table legend, main text, or Methods section.

n/a	Confirmed	
☐	☒	The exact sample size (n) for each experimental group/condition, given as a discrete number and unit of measurement
☐	☒	A statement on whether measurements were taken from distinct samples or whether the same sample was measured repeatedly
☐	☒	The statistical test(s) used AND whether they are one- or two-sided<br>*Only common tests should be described solely by name; describe more complex techniques in the Methods section.*
☐	☒	A description of all covariates tested
☒	☐	A description of any assumptions or corrections, such as tests of normality and adjustment for multiple comparisons
☐	☒	A full description of the statistical parameters including central tendency (e.g. means) or other basic estimates (e.g. regression coefficient) AND variation (e.g. standard deviation) or associated estimates of uncertainty (e.g. confidence intervals)
☐	☒	For null hypothesis testing, the test statistic (e.g. F, t, r) with confidence intervals, effect sizes, degrees of freedom and P value noted *Give P values as exact values whenever suitable.*
☒	☐	For Bayesian analysis, information on the choice of priors and Markov chain Monte Carlo settings
☒	☐	For hierarchical and complex designs, identification of the appropriate level for tests and full reporting of outcomes
☐	☒	Estimates of effect sizes (e.g. Cohen's d, Pearson's r), indicating how they were calculated

Our web collection on statistics for biologists contains articles on many of the points above.

Software and code

Policy information about availability of computer code

Data collection	*Provide a description of all commercial, open source and custom code used to collect the data in this study, specifying the version used OR state that no software was used.*
Data analysis	*Provide a description of all commercial, open source and custom code used to analyse the data in this study, specifying the version used OR state that no software was used.*

For manuscripts utilizing custom algorithms or software that are central to the research but not yet described in published literature, software must be made available to editors/reviewers. We strongly encourage code deposition in a community repository (e.g. GitHub). See the Nature Research guidelines for submitting code & software for further information.

Data

Policy information about availability of data

All manuscripts must include a data availability statement. This statement should provide the following information, where applicable:

- Accession codes, unique identifiers, or web links for publicly available datasets
- A list of figures that have associated raw data
- A description of any restrictions on data availability

Relevant microarray data has been uploaded GSE104740. The mass spectrometry proteomics data have been deposited to the ProteomeXchange Consortium via the PRIDE [1] partner repository with the dataset identifier PXD014736.

Field-specific reporting

Please select the one below that is the best fit for your research. If you are not sure, read the appropriate sections before making your selection.

☒ Life sciences ☐ Behavioural & social sciences ☐ Ecological, evolutionary & environmental sciences

For a reference copy of the document with all sections, see nature.com/documents/nr-reporting-summary-flat.pdf

Life sciences study design

All studies must disclose on these points even when the disclosure is negative.

Sample size	No sample size calculation was performed.<br>The number of cell lines used in this study was determined by the mesenchymal signature of the cell lines.<br>Given the consistency in the results across cell lines we believe our sample size was sufficient.<br>For the interluminal experiments, the number of mice per group was determined based on tumour growth by luciferase. Permitting valid statistical significance confirming the adequacy of the sample size.<br>For the intermural experiments, the number of mice per group was determined based on tumour growth by luciferase. Permitting valid statistical significance confirming the adequacy of the sample size.
Data exclusions	No data was excluded from the analysis.
Replication	All experiments were successfully reproduced (excluding occasional failures due to technical errors).
Randomization	For determination of tumour growth mice were randomly treated with inhibitor of control.
Blinding	Technicians and researchers were blinded to group allocation during data collection (tumor measurement).

Reporting for specific materials, systems and methods

We require information from authors about some types of materials, experimental systems and methods used in many studies. Here, indicate whether each material, system or method listed is relevant to your study. If you are not sure if a list item applies to your research, read the appropriate section before selecting a response.

Materials & experimental systems

n/a	Involved in the study	
☐	☒	Antibodies
☐	☒	Eukaryotic cell lines
☒	☐	Palaeontology
☐	☒	Animals and other organisms
☒	☐	Human research participants
☒	☐	Clinical data

Methods

n/a	Involved in the study	
☒	☐	ChIP-seq
☒	☐	Flow cytometry
☒	☐	MRI-based neuroimaging

Antibodies

Antibodies used	E-cadherin (BD), anti-FLAG and anti-α-tubulin (Sigma), anti-HA (Y11, Santa-Cruz biotech), anti-Myc (9E10 or A14, Santa-Cruz Biotech), anti-phospho-SMAD2 (S465/467, Cell Signaling), anti-Laminin-b1 (Abcam), anti-SMAD2 (L16D3, Cell Signaling), anti-β-actin (Sigma), anti-TGF-β receptor I (V-22, Santa-Cruz Biotech), anti-phospho-tyrosine (clone 4G10, Millipore, 05-321), anti-Phospho-Src (Tyr 416, 2101S, Cell Signaling), anti-c-Src (B-12, Santa Cruz Biotech), anti-phospho-ERK (P-p44/42 MAPK T202/Y204, 9101, Cell Signaling), anti-Total ERK (p44/42 MAPK, 9102, Cell Signaling), anti-p38 (Cell Signaling), p-c-MET Tyr1234/1235 Cell Signalling), and anti-phospho-SMAD2 (S245/250/255, 3104, Cell Signaling). The pan TGFβ neutralizing antibody (ID11) was purchased from Genzyme, the activin ligand trap soluble ACVR2B-Fc was a kind gift from Olli Ritvos (University of Helsinki, Finland).
Validation	Where possible, the antibodies were validated for western blot by using knockout cells as negative controls.<br>All antibodies were used at 1:1000 dilution for western blot studies.

Eukaryotic cell lines

Policy information about cell lines

Cell line source(s)	All cell lines were purchased from ATCC. NBT-II cell lines have been used in our lab for the last 30 years.
Authentication	Cell lines were autheticated using Genemapper from Applied biosystems.
Mycoplasma contamination	All the cell lines were tested negative for mycoplasma contamination
Commonly misidentified lines (See ICLAC register)	No commonly misidentified cell lines were used.

Animals and other organisms

Policy information about studies involving animals; ARRIVE guidelines recommended for reporting animal research

Laboratory animals	6 to 8 weeks old-female balb/c nude (nu/nu) mice were used
Wild animals	N/A
Field-collected samples	N/A
Ethics oversight	All animal work adhered to the Agency of Science Technology and Research (A*STAR), Institutional Animal Care and use Committee (IACUC), guidelines on animal use and handling.

Note that full information on the approval of the study protocol must also be provided in the manuscript.

This checklist template is licensed under a Creative Commons Attribution 4.0 International License, which permits use, sharing, adaptation, distribution and reproduction in any medium or format, as long as you give appropriate credit to the original author(s) and the source, provide a link to the Creative Commons license, and indicate if changes were made. The images or other third party material in this article are included in the article's Creative Commons license, unless indicated otherwise in a credit line to the material. If material is not included in the article's Creative Commons license and your intended use is not permitted by statutory regulation or exceeds the permitted use, you will need to obtain permission directly from the copyright holder. To view a copy of this license, visit http://creativecommons.org/licenses/by/4.0/
